# Can Esketamine Become a Viable Alternative for Treating Treatment-Resistant Depression in Ecuador?

**DOI:** 10.1192/j.eurpsy.2025.1353

**Published:** 2025-08-26

**Authors:** E. Pazmiño, K. Armas

**Affiliations:** 1 Psiquiatría, Instituto Psiquiátrico Sagrado Corazón; 2 Psiquiatría, Hospital Metropolitano; 3 Medicina Interna, Clínica Pasteur, Quito, Ecuador

## Abstract

**Introduction:**

Treatment-resistant Depression (TRD) nowadays it is consider a public health problem. Studies had demonstrate that TRD has higher prevalence of psychiatric comorbid conditions, twice the utilization of outpatient health care resources, 3 times the number of inpatient bed-days, and 23% higher all-cause mortality. Esketamine, the *S*-enantiomer of ketamine, has been recently approved for depression that has failed to respond to two or more antidepressants ^2^. Nevertheless, considering its steep cost, accessibility in Ecuador becomes a crucial factor. Comprehensive studies are essential to substantiate its efficacy

**Objectives:**

Evaluate the effectiveness and safety of esketamine nasal spray in a clinical sample of patients with TRD

**Methods:**

This is an observational, retrospective and multicentric study comprising a total of 16 TRD patients treated with esketamine nasal pray, the sample was collected over a period of 2 years. Anamnestic data and psychometric assessment (MADRS and Columbia scale for suicidal ideation) were collected from medical records at baseline (T0), one month (T1) and two month (T2) follow-ups.

**Results:**

Clinical response was achieved in 68% at T1 and 81% by T2. Remission rates of 50% was detected by T2. Few side effects were seen in this study, 25% (4) present disossiacion 13% (3) hypertension and anxiety 12% (2) all of them were autolimited and no treatment was required. Based on the Columbia scale to assess suicidal ideation, the disappearance of suicidal risk was observed before T1 in all cases. It is important to emphasize that 2 of the patients taken into account for the study abandoned treatment before the time established. The first patient abandoned it in the second application for economic reasons and the second patient because he observed total remission of the symptoms by T1

**Conclusions:**

Taking into account the results, it can be concluded that esketamine is a safe medication, given the low percentage of observed adverse effects, all of which were mild and self-limiting. Moreover, the high rates of clinical response and remission allow us to conclude its effectiveness. However, the restriced accessibility should be taken into acount due to the elevated cost of esketamine which also limits this study due to the small sample size

**Disclosure of Interest:**

None Declared

